# Trends and Prospects in Sustainable Food Packaging Materials

**DOI:** 10.3390/foods13111744

**Published:** 2024-06-01

**Authors:** Wenya Ma, Li Li

**Affiliations:** College of Food Science and Technology, Shanghai Ocean University, Shanghai 201306, China; d220300078@st.shou.edu.cn

## 1. Introduction

Food packaging plays an important role in delaying the spoilage of fresh food during transportation and storage. The high annual demand for food packaging exerts significant environmental pressure. Sustainable food packaging is essential for reducing waste and protecting the environment. The degradable plastics available in the market can be categorized into two primary types: “petroleum-derived” and “biobased” polymers [1]. These polymers serve as the primary components of packaging, contributing to its fundamental mechanical and barrier properties. The biobased substrates include polysaccharides, proteins, and lipids. A combination of these materials with functional ingredients such as plant extracts and nanoparticles is commonly employed to achieve functions such as antioxidant, antimicrobial, and antifogging properties, as well as to regulate the microenvironment for improved food preservation and freshness [2,3,4]. Various modification methods can also enhance the performance of films, such as the surface plasma, crosslinking, blend, grafting, and corona treatment [5,6]. There are different film formation methods for biopolymers, such as the solution casting method, melt extrusion, electrospinning method, hot-pressing, casting, coating, and extrusion blown film method. Different preparation processes are selected according to the characteristics of the substrate to improve the quality, healthiness, and sustainability of the packaging in food supply chains.

## 2. An Overview of Published Articles

This Special Issue is composed of six different works, five research papers and one review, provided by researchers in the field of food packaging, which highlight the effects and applications of sustainable food packaging in different types of foods.

The article by Deng et al. (contribution 1) is titled “Effect of Surfactant Formula on the Film Forming Capacity, Wettability, and Preservation Properties of Electrically Sprayed Sodium Alginate Coats”. In this study, the effects of the mix surfactants Tween 20 and Span 80, with different hydrophile–lipophile balance values, on the film-forming ability, wettability, and preservation capacity of blueberry sodium alginate coating were investigated. The sodium alginate coating with low viscosity and a medium hydrophile-lipophile balance displayed a superior coating performance, which could better inhibit the metabolism of blueberries, and thus repressed the degradation to reduce the loss of blueberry quality. The hydrophile–lipophile balance was crucial for the preservation effects of edible coatings and needed to be accurately designed when determining the film-forming formula. This work provides a valuable reference for the selection of surfactants on coating systems.

The article by Liu et al. (contribution 2) prepared the pH-responsive films from polyvinyl alcohol/agar containing cochineal to monitor the freshness of pork. As the pH of the cochineal solution ranged from 2 to 6 and 12, the color changed from orange to red, and subsequently to purple. The cochineal also provided good antioxidant and antibacterial effects to the film. The polyvinyl alcohol/agar film containing cochineal applied to pork exhibited color changes from orange to dark purple, indicating the spoilage of pork. The pH-responsive films, which track the freshness of protein-rich fresh food in a non-destructive way, have great market potential in terms of their functionality and preservation efficacy.

The article by Ciano et al. (contribution 3) provided an analysis of the substitute materials and their applications in food contact materials in the Belgian market through a typical market investigation. This survey offered valuable insights for policymakers and researchers in the field of alternative materials. The results showed that paper and paper analogues dominated the substitute materials market, while wood analogues also exhibited promising potential. The findings can also provide valuable guidance for waste reduction, recycling, and the promotion of sustainable development for other regions.

The fourth text published in this Special Issue is a review by Li et al. (contribution 4) on the recent advances in intelligent packaging aided by artificial intelligence for monitoring food freshness. This work summarized the research progress and outlined the advantages and disadvantages of using intelligent packaging technology to detect food freshness. Artificial intelligence could assist and strengthen intelligent packaging technology, allowing for higher efficiency in food freshness detection. The authors provide valuable insights for the future development of intelligent packaging in the field of food freshness detection.

The article by Ding et al. (contribution 5) developed a biodegradable, gas-regulating packaging film (PLA/PBAT/TPS-MCSA) by incorporating the natural plant growth regulator, salicylic acid, into MCM-41, and its application was extended to the preservation of bananas. A comparative analysis revealed that the PLA/PBAT/TPS-MCSA film, exhibited a superior performance compared to PLA/PBAT/TPS, and PLA/PBAT/TPS-SA films. It delayed the rate of weight loss and increase of malondialdehyde while inhibiting the polyphenol oxidase activity, thereby prolonging the shelf life of bananas by 4–5 days. The innovative biodegradable packaging developed in this research provides a promising approach to the future gas-regulated packaging of fruits and vegetables.

Research by Fronza et al. (contribution 6) developed a bio-nanocomposite based on locust bean galactomannan and the cassava waste of starch and cellulose nanofibers. The raw materials used for preparing the film were all derived from the waste of cassava and locust. The utilization of waste-derived raw materials for film preparation can effectively reduce agricultural waste and enhance the value-added utilization of such resources. The prepared films have great mechanical, barrier, chemical, and structural properties. Moreover, the bio-nanocomposite remained stable in acidic and alkaline pH conditions during a 12-day test period. The film was biodegradable within five days, contributing to a reduction in the environmental impacts caused by the improper disposal of cassava waste and synthetic packaging. The article is a typical example of using waste materials to produce sustainable packaging films.

## 3. Future Trends and Conclusions

Packaging materials prepared from biopolymer sources have gained significant popularity among consumers and on the market due to their eco-friendly nature. As sustainability becomes a growing concern, the safety of packaging materials has also received more attention. Sustainable packaging still encounters numerous challenges, necessitating further enhancements. Firstly, it is crucial to ensure that the active ingredients in the packaging maintain their longevity and effectiveness for an extended period. Therefore, methods and techniques for the long-acting release of active ingredients within the films are needed. Another challenge in sustainable food packaging is controlling the production costs. Improving the processing and utilization of agricultural by extracting valuable substances from agricultural products, including fruit residues like pineapple peels and dragonfruit peels, is a great way to reduce production costs. Many previous studies also reported that packaging films prepared by blending the extract of waste materials with polymers show an excellent performance [7,8]. Edible films and coatings are important categories of sustainable food packaging. The benefits and risks of edible packaging to human health remain controversial. The preparation process and raw materials used in the edible packaging need to meet higher requirements to ensure both edible performance and packaging performance, such as mechanical properties and heat-sealing. There is a growing demand for multi-functional food packaging that can cater to the various needs of foods during transportation, storage, and sales processes.

In summary, the development of sustainable food packaging materials holds great significance for the preservation of food and the protection of the environment. At the same time, it also helps to promote the high-value utilization of biological bases. The advancement of sustainable packaging requires the adoption of more innovative packaging technologies.

## Data Availability

No new data were created or analyzed in this study. Data sharing is not applicable to this article.
